# Uncommon association of coronary artery ectasia and myocardial bridge presenting as non–ST-segment elevation myocardial infarction: a case report

**DOI:** 10.3389/fcvm.2025.1727448

**Published:** 2025-12-09

**Authors:** Marlon Rojas-Cadena, Felipe Rodríguez-Arcentales, Colon Arteaga, Sylvia Davila, Juan Carlos Gaibor, Juan S. Izquierdo-Condoy

**Affiliations:** 1 One Health Research Group, Faculty of Medicine, Universidad de las Américas, Quito, Ecuador; 2 Department of Cardiology, Hospital de Especialidades Carlos Andrade Marín, Quito, Ecuador; 3 Department of Cardiology, Hospital Metropolitano, Quito, Ecuador

**Keywords:** coronary artery ectasia, myocardial bridging, NSTEMI, antithrombotic therapy, conservative management

## Abstract

**Introduction:**

Coronary artery ectasia (CAE)—diffuse dilatation ≥1.5× the adjacent segments is uncommon and lacks standardized management. Its coexistence with a hemodynamically significant myocardial bridge (MB) is unusual and may create competing disturbances in coronary flow that complicate diagnosis and treatment.

**Case presentation:**

An 80-year-old man with hypothyroidism, epilepsy, benign prostatic hyperplasia, and paroxysmal atrial fibrillation on rivaroxaban presented with acute precordial pain consistent with non–ST-segment elevation myocardial infarction (NSTEMI). He was hemodynamically stable; ECG showed inferior ST depression with T-wave inversion in V3–V4, and high-sensitivity troponin was elevated (Killip I, GRACE 177, CRUSADE 49). Early diagnostic angiography (<24 h) revealed diffuse three-vessel ectasia (Markis I) with slow TIMI-2 flow and a prominent mid-LAD MB (∼75% systolic “milking”); the intermediate branch had an ostial lesion with downstream aneurysmal dilatation and was not amenable to PCI. Echocardiography showed LVEF >65% with basal inferior/inferoseptal hypokinesia and severe left-atrial enlargement (57 ml/m^2^). A diagnosis of type 2 NSTEMI due to supply–demand mismatch in the setting of diffuse CAE and MB was established. He was treated with clopidogrel (single antiplatelet therapy) (INR 2.0–3.0), high-intensity statin, and beta-blocker, with symptomatic improvement and remained asymptomatic without recurrent ischemic events over a 4-month follow-up.

**Conclusions:**

Diffuse CAE with significant MB can precipitate NSTEMI without discrete obstructive lesions and challenges standard revascularization. In such anatomy, individualized conservative therapy—rate control and tailored antithrombotic management—may be preferable, while advanced imaging and diastolic physiology can refine diagnosis and selection for invasive strategies.

## 1 Introduction

Coronary artery ectasia (CAE) is defined as a diffuse dilatation of a coronary segment to at least 1.5 times the diameter of an adjacent normal segment; by contrast, “coronary artery aneurysm” (CAA) is typically used for focal dilatations, whereas CAE refers to more diffuse involvement (1). The reported prevalence of CAE varies by geography and population, ranging from ∼0.3% to 5% in angiographic series, with a male predominance; the right coronary artery (RCA) is most commonly affected (2, 3). Clinically, CAE presents heterogeneously—from stable angina to acute coronary syndromes (ACS), and even atypical anginal equivalents—reflecting a pathophysiologic substrate of sluggish flow, distal embolization, and thrombosis within ectatic segments (3). Although multiple mechanisms have been proposed (atherosclerosis, inflammatory and connective-tissue disorders), a standardized management algorithm is lacking (4). Prognosis is likewise variable, with observational data suggesting higher rates of adverse events, including no-reflow and stent thrombosis when percutaneous coronary intervention (PCI) is attempted in markedly ectatic or aneurysmal vessels (4).

Myocardial bridging (MB)—systolic compression of a tunneled coronary segment, most often the mid–left anterior descending (LAD) artery—is a relatively common anatomic variant. It is detected more frequently by coronary computed tomography angiography (CCTA) than by invasive angiography (approximately 19%–22% vs. 2%–6%) and can be associated with angina, ACS, arrhythmias, and, rarely, sudden death, particularly under tachycardic states that curtail diastolic perfusion (4, 5). The coexistence of diffuse CAE and a hemodynamically significant MB is unusual and may create competing perturbations of coronary flow—stasis and thrombus formation within ectatic segments, with dynamic systolic compression over the bridged segment—complicating both diagnosis and treatment strategies.

## 2 Case presentation

We report the case of an 80-year-old man with a medical history of hypothyroidism, epilepsy, benign prostatic hyperplasia, paroxysmal atrial fibrillation, and vertigo. He was independent in his daily activities and had good adherence to his prescribed medications. He reported no relevant family medical history. Chronic medications included lamotrigine, clonazepam, tamsulosin, dutasteride, and rivaroxaban 20 mg daily.

He presented to the emergency department with acute precordial chest pain (8/10 on the visual analog scale) radiating to the left shoulder, without vasovagal symptoms. On admission, he was hemodynamically stable: heart rate 71 beats/min (regular rhythm), blood pressure 106/61 mmHg, and oxygen saturation 90% on room air. Physical examination revealed no signs of cardiopulmonary or hemodynamic instability. The initial electrocardiogram (ECG) showed ST-segment depression in the inferior leads and T-wave inversion in V3–V4, consistent with myocardial ischemia. High-sensitivity cardiac troponin was elevated, confirming non–ST-segment elevation myocardial injury compatible with an NSTEMI presentation. He was Killip class I, with a CRUSADE bleeding score of 49 and a GRACE score of 177 (high ischemic risk). Admission laboratory parameters—including hs-troponin-T 0.03 ng/ml, NT-proBNP 1,658 pg/ml, creatinine 0.96 mg/dl, LDL-C 88 mg/dl, HDL-C 38 mg/dl, triglycerides 192 mg/dl, and HbA1c 6.04%—are summarized in Table 1.

**Table 1:** Clinical–hematological profile of the patient.

Test	Results	Reference range
WBC	4.86 × 10^3^/μl	3.4–9.7 × 10^3^/μl
Neutrophil	2.48 × 10^3^/μl	2.2–4.8 × 10^3^/μl
Lymphocyte	1.62 × 10^3^/μl	1.1–3.2 × 10^3^/μl
Hemoglobin	15 g/dl	14.0–18.0 g/dl
Platelet	161,000/μl	130,000–400,000/μl
Troponin T	0.03 ng/ml	0–0.01 ng/ml
Pro BNP	1,658 pg/ml	<125 pg/ml
Creatinin	0.96 mg/dl	0.74–1.35 mg/dl
LDL	88 mg/dl	<100 mg/dl
HDL	38 mg/dl	40–59 mg/dl
Triglycerides	192 mg/dl	<150 mg/dl
HbA1c	6.04%	4–5, 6%

Values are from admission laboratory testing. WBC, white blood cell count; NT-proBNP, N-terminal pro–B-type natriuretic peptide; LDL-C, low-density lipoprotein cholesterol; HDL-C, high-density lipoprotein cholesterol; HbA1c, glycated hemoglobin. Reference ranges according to the institutional laboratory.

Given the high-risk ECG pattern and biomarker elevation, an early invasive strategy with diagnostic coronary angiography was undertaken within the first 24 h; percutaneous coronary intervention (PCI) was not performed due to the angiographic substrate. Before angiography, he received guideline-directed medical therapy, including dual antiplatelet therapy and high-intensity statin.

**Figure 1:**
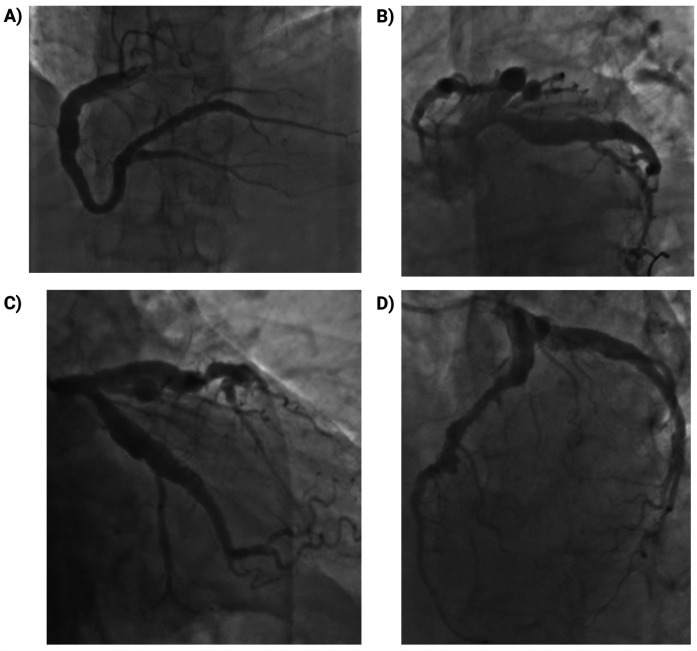
Coronary angiography showing diffuse multivessel coronary ectasia (markis type I). **(A)** Right coronary artery (RCA): marked ectasia with slow flow (TIMI 2); maximum reference diameter ∼8.7 mm; diffuse non-obstructive atherosclerosis. **(B)** Left main coronary artery (LMCA): ectatic with markedly enlarged ostium and trunk; no intraluminal lesions. **(C)** Left anterior descending artery (LAD): marked ectasia with diffuse atheroma; maximum reference diameter ∼7.1 mm. **(D)** Left circumflex artery (LCx): ectatic, large-caliber vessel with slow flow (TIMI 2); maximum reference diameter ∼7.2 mm; side branches with diffuse non-obstructive plaques.

**Figure 2:**
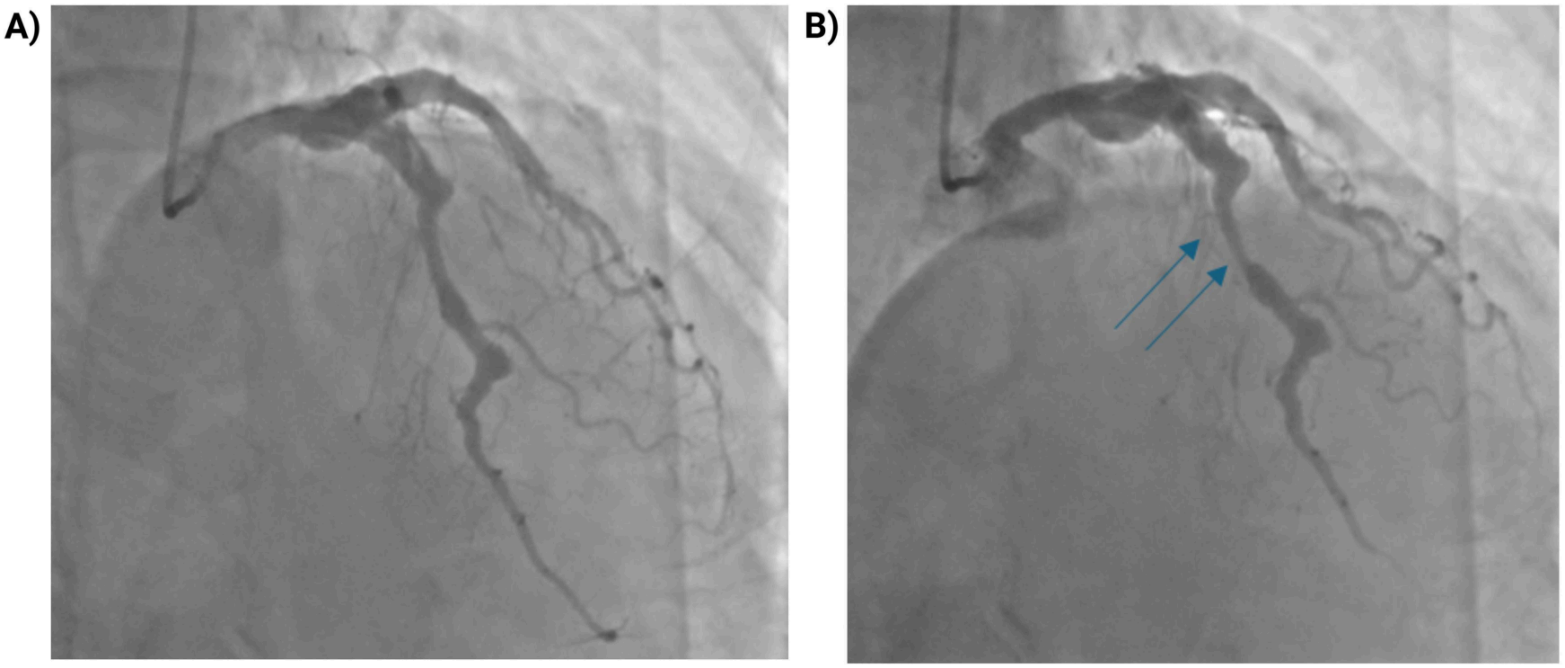
Myocardial bridging of the mid-LAD with dynamic systolic compression. **(A)** Coronary angiogram in diastole showing the mid-LAD segment. **(B)** During systole, dynamic compression of a myocardial bridge in the mid-LAD segment (blue arrows) produces a “milking effect” with approximately 75% systolic narrowing. The apparent degree of stenosis may be overestimated due to the surrounding coronary ectasia.

Coronary angiography demonstrated diffuse coronary ectasia involving all major epicardial vessels (Markis type I) with slow TIMI 2 flow. Maximal reference diameters exceeded 8.7 mm in the right coronary artery, 7.2 mm in the circumflex, and 7.1 mm in the left anterior descending (LAD) artery (Figure 1). The mid-LAD showed a prominent myocardial bridge (MB) with a characteristic “milking effect” (∼75% systolic compression), likely accentuated by the surrounding ectatic reference segment (Figure 2). The intermediate branch displayed a severe ostial lesion followed by aneurysmal dilatation (maximum diameter 6.5 mm) and was deemed unsuitable for PCI. Diffuse, non-obstructive atherosclerotic plaques were present throughout the coronary tree without significant fixed stenoseS.

Based on the findings observed on coronary angiography, a Type 2 myocardial infarction secondary to supply–demand mismatch was established. The markedly slow and turbulent flow in the ectatic segments, combined with the hemodynamically significant myocardial bridge which further increased dynamic obstruction and impaired distal perfusion—supported this diagnosis.

Transthoracic echocardiography showed a preserved left ventricular ejection fraction (>65%). Regional wall-motion abnormalities were noted (hypokinesia of the basal inferior and basal inferoseptal segments). Diastolic function was grade I (mild). Right ventricular systolic function was normal [tricuspid annular plane systolic excursion (TAPSE) 20 mm]. The left atrium was severely enlarged (volume index 57 ml/m^2^). No significant valvular abnormalities were identified, and the pericardium was normal.

Given the high bleeding risk and the extensive ectatic anatomy without focal, flow-limiting stenosis, single antiplatelet therapy (SAPT) with clopidogrel was preferred, and anticoagulation was continued with warfarin (target INR 2.0–3.0). In line with contemporary recommendations for patients requiring long-term oral anticoagulation without recent stent implantation, we selected clopidogrel as the single antiplatelet agent and withheld aspirin to minimize major bleeding risk while maintaining adequate platelet inhibition. A high-intensity statin and a beta-blocker were also administered, and his pre-existing medications were maintained. He responded favorably to medical therapy with symptom improvement and was discharged in stable condition.

**Figure 3:**
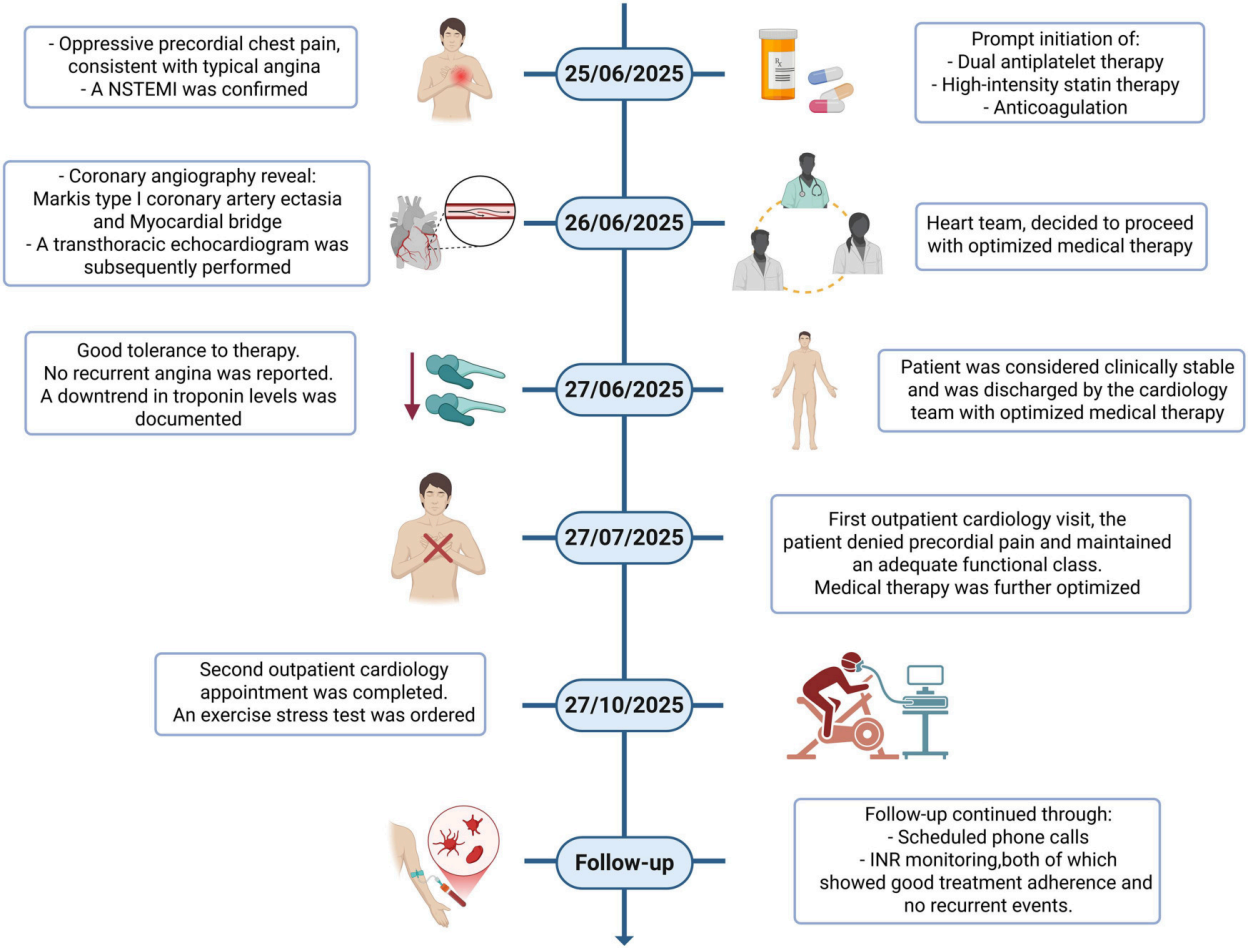
Chronological summary of the clinical course. Timeline illustrating symptom onset, emergency department presentation, ECG changes, biomarker trajectory, timing of coronary angiography, in-hospital management, discharge, and outpatient follow-up visits.

During follow-up, the patient was evaluated twice by the cardiology department: the first visit focused on optimizing medical therapy, and the second involved ordering an exercise stress test as part of the entry assessment for the cardiac rehabilitation program. Additional follow-up was conducted through scheduled phone calls and INR monitoring, both of which demonstrated good treatment adherence and no recurrent events. The first in-person visit took place one month after hospital discharge, and the second occurred three months later; the latter represented the final in-person contact of a four-month follow-up period. Throughout this interval, the patient remained asymptomatic, and no repeat coronary angiography or CCTA was deemed necessary given the absence of recurrent ischemic symptoms and stable laboratory parameters apart from routine INR monitoring (Figure 3).

## 3 Discussion

CAE exhibits a wide clinical spectrum. Some patients present with concomitant, functionally significant stenoses, whereas others—like ours—show diffuse ectasia without fixed critical obstruction. Presentations range from typical angina to ACS, including both STEMI and NSTEMI, driven by a combination of slow-flow/no-reflow, *in situ* thrombosis, and distal embolization (6). Most CAE in adults is acquired and associated with atherosclerosis; traditional risk factors include hypertension, smoking, and familial hypercholesterolemia, while less common etiologies comprise inflammatory and connective-tissue disorders (e.g., vasculitides, systemic lupus erythematosus, Ehlers–Danlos, and Kawasaki disease) (2).

Myocardial bridging (MB) is a relatively common anatomical variant whose true prevalence depends on the diagnostic modality—higher in autopsy series and CCTA, and lower by invasive angiography. The LAD—especially its proximal and mid segments—is most frequently involved; the LCx and RCA are less often affected. CCTA detects MB more often than conventional angiography (∼19%–22% vs. 2%–6%) (7). Physiologically, MB can precipitate supply–demand mismatch by accentuating systolic compression and abbreviating diastolic perfusion during tachycardia. Depth, length, and location of a MB all contribute to the severity of vessel compression; the duration of systolic narrowing is also relevant. In general, a MB is considered hemodynamically significant when there is a visually estimated ≥70% reduction in the minimal luminal diameter during systole, with ≥35% persistent narrowing extending into early diastole (7, 8). In our patient, angiography documented a prominent mid-LAD bridge with ∼75% systolic “milking effect”, superimposed on Markis type I three-vessel ectasia with TIMI 2 slow flow—two substrates that synergistically impair effective myocardial perfusion despite the absence of a discrete, focal culprit stenosis. Although the regional wall-motion abnormality was confined to the basal inferior and inferoseptal segments, which correspond predominantly to the right coronary artery territory, this finding is consistent with the markedly ectatic RCA and globally impaired TIMI 2 flow in all three epicardial vessels. We therefore interpret the event as the result of a combined mechanism—diffuse Markis type I ectasia with slow flow, particularly in the RCA, together with a hemodynamically significant mid-LAD bridge—rather than a single-vessel culprit lesion.

Revascularization in ectatic/aneurysmal culprit vessels is technically challenging and carries higher risks of no-reflow, distal embolization, and stent thrombosis; intermediate-term outcomes after PCI in this context include increased mortality, target vessel revascularization, and recurrent MI (1). Mechanistically, device sizing and apposition are problematic in markedly dilated segments; dense thrombus burden and sluggish flow favor acute/subacute thrombosis; and, when MB coexists, dynamic compression adds mechanical stress that may predispose to stent fracture or restenosis if a device is implanted across the bridged segment (9). Accordingly, in the absence of a focal, flow-limiting lesion, contemporary management often prioritizes optimized medical therapy with individualized antithrombotic regimens (4).

Evidence guiding antithrombotic strategy in CAE is limited. A small randomized trial in ACS suggested that DOAC plus SAPT reduces bleeding vs. DAPT while preserving ischemic protection (10). Notably, our patient experienced an ischemic event despite chronic rivaroxaban for atrial fibrillation. In this setting—diffuse Markis I ectasia, angiographic slow flow, and prior DOAC exposure at the time of presentation—the heart-team favored SAPT plus warfarin with INR monitoring to balance thrombotic and bleeding risks and to allow tight anticoagulation control (11). Case-based experience has likewise reported favorable outcomes with warfarin-based regimens in recurrent ischemia related to ectasia, though high-quality comparative data are lacking (11). Given the coexistence of MB, beta-blockade was instituted to lower heart rate and contractility, prolong diastole, and lessen systolic compression; nitrates were avoided due to the potential to exacerbate dynamic narrowing via reflex tachycardia and increased vessel compliance (5). High-intensity statin therapy was prescribed to target shared atherothrombotic risk factors.

Diagnostic interpretation in CAE with MB merits nuance. First, angiography can overestimate the apparent percent stenosis adjacent to ectatic reference segments, which can mislead revascularization decisions. Second, although invasive coronary angiography established the diagnosis in our case, CCTA offers superior anatomic delineation of MB and aneurysmal/ectatic morphology and may be preferred when the clinical context allows (7). Third, intravascular imaging (e.g., IVUS) improves diagnostic precision in aneurysmal/ectatic segments and can clarify plaque/thrombus composition; it was not available in our setting. Advanced intracoronary imaging (IVUS/OCT) and CCTA were not available in our institution at the time of presentation, which limited further anatomic and functional characterization and represents an important limitation of this report. Finally, when symptoms persist despite optimal therapy, functional assessment focused on diastolic physiology (e.g., diastolic FFR/iFR) may help quantify bridging significance and guide consideration of advanced therapies (9).

Our case complements the limited literature on the coexistence of CAE/CAA and MB, as summarized in Table 2. Czepe et al. reported CAE with MB diagnosed by CCTA but did not detail management, underscoring the variability in diagnostic pathways (9). Zhen Ye et al. described CAA with MB characterized by angiography and IVUS and managed medically with antiplatelet and anti-ischemic therapy, with favorable follow-up (12). In contrast, our patient presented with NSTEMI on a background of diffuse Markis I ectasia and a hemodynamically significant mid-LAD bridge; the combination of extensive ectasia, slow flow, and dynamic compression—without a discrete, stentable culprit—supported a conservative strategy of SAPT plus oral anticoagulation and rate-control therapy.

**Table 2:** Summary of previous case reports of coronary ectasia plus myocardial bridging.

Reported cases	Sex/Origin	Age at presentation	Type of coronary ectasia	Diagnostic test/method	Treatment	Outcomes
Case 1. Ye Z. et al., 2021 (12)	Woman, Fujian Province, China	54-year-old	Markis type III	Coronary Angiography (CAG)	Aspirin 100 mg/d, Rosuvastatin 10 mg/d, Metoprolol 47.5 mg/d, and Amlodipine 5 mg/d	The patient was in good condition at the 5-mo follow-up by phone.
Case 2. Czepe G. et al., 2024 (9)	Woman, Lublin, Poland	78-year-old	Markis type III	Coronary computed tomography angiography (CCTA),	No specific description. Currently: clinical observation at a cardiology outpatient clinic, measures to modify risk factors and optimize treatment, including treatment of hypertension.	There is no description of the follow-up.
Current report	Man, Quito, Ecuador	80-year-old	Markis type I	Coronary angiography (CAG)	Clopidogrel 75 mg/d, warfarin (INR 2.0–3.0), high-intensity statin, beta-blocker, plus standard therapy for comorbidities.	Asymptomatic with no recurrent ischemic events during 4-month follow-up; therapeutic INR on serial monitoring.

CAG, coronary angiography; CCTA, coronary computed tomography angiography; INR, international normalized ratio; d, day; mo, month.

Interventional or surgical therapies should be reserved for selected refractory cases—for example, deep or long bridges (>25 mm length or >5 mm depth), objective ischemia persisting despite optimized medical therapy, or when a discrete, flow-limiting stenosis is demonstrable (9). PCI across MB carries non-trivial risks, including in-stent restenosis and stent fracture; surgical options (myotomy or CABG) are considered when symptoms and ischemia remain refractory, but myotomy bears risks such as ventricular perforation, aneurysm formation, and bleeding (9). In our patient, interventional therapy was not pursued given the absence of a clear PCI target, the unfavorable ectatic substrate, and the favorable clinical response to medical therapy (4, 5, 11).

Finally, while CAE and CAA are distinct entities—diffuse vs. focal dilation (≥1.5× the reference diameter)—they share etiologies and pathophysiologic mechanisms and are sometimes conflated in clinical practice. To our knowledge, reports of myocardial infarction in the setting of Markis I CAE coexisting with MB remain scarce; our case underscores that this coexistence may be under-recognized in ACS and that outcomes can be acceptable with carefully individualized, conservative management. During follow-up, our patient remained asymptomatic, with therapeutic INR values and no recurrent ischemic events.

## 4 Conclusions

Diffuse coronary artery ectasia (Markis type I) coexisting with a hemodynamically significant mid-LAD myocardial bridge can precipitate NSTEMI without discrete obstructive lesions and challenges conventional revascularization strategies. In this anatomic context, PCI may be hazardous (thrombus burden, malapposition, no-reflow, device complications), making optimized medical therapy—rate control with beta-blockade, high-intensity statin, and individualized antithrombotic therapy—a reasonable first-line approach.

This case also highlights interpretive pitfalls of angiography (overestimation of stenosis adjacent to ectatic segments) and the potential value of CCTA/IVUS and diastolic physiologic assessment when available. With careful risk–benefit appraisal, avoidance of nitrates in MB, and structured follow-up (including INR monitoring), favorable outcomes are achievable, while registries and comparative studies are needed to refine antithrombotic selection and indications for invasive or surgical treatment in CAE with concomitant MB.

## Data Availability

The original contributions presented in the study are included in the article/Supplementary Material, further inquiries can be directed to the corresponding author.
